# Activation of BDNF by transcription factor Nrf2 contributes to antidepressant-like actions in rodents

**DOI:** 10.1038/s41398-021-01261-6

**Published:** 2021-02-24

**Authors:** Wei Yao, Song Lin, Jin Su, Qianqian Cao, Yueyue Chen, Jiaxu Chen, Zhentao Zhang, Kenji Hashimoto, Qi Qi, Ji-chun Zhang

**Affiliations:** 1 Formula-Pattern Research Center, School of Traditional Chinese Medicine, Jinan University, 510632 Guangzhou, China; 2 Department of Physiology, School of Medicine, Jinan University, 510632 Guangzhou, China; 3 MOE Key Laboratory of Tumor Molecular Biology; Clinical Translational Center for Targeted Drug, Department of Pharmacology, School of Medicine, Jinan University, 510632 Guangzhou, China; 4 Guangzhou Overseas Chinese Hospital of Jinan University, 510632 Guangzhou, China; 5 Department of Neurology, Renmin Hospital of Wuhan University, 430060 Wuhan, China; 6 Division of Clinical Neuroscience, Chiba University Center for Forensic Mental Health, Chiba, 260–8670 Japan

**Keywords:** Depression, Molecular neuroscience

## Abstract

The transcription factor erythroid 2-related factor 2 (Nrf2) and brain-derived neurotrophic factor (BDNF) play a key role in depression. However, the molecular mechanisms underlying the crosstalk between Nrf2 and BDNF in depression remain unclear. We examined whether Nrf2 regulates the transcription of *Bdnf* by binding to its exon I promoter. Furthermore, the role of Nrf2 and BDNF in the brain regions from mice with depression-like phenotypes was examined. Nrf2 regulated the transcription of *Bdnf* by binding to its exon I promoter. Activation of Nrf2 by sulforaphane (SFN) showed fast-acting antidepressant-like effects in mice by activating BDNF as well as by inhibiting the expression of its transcriptional repressors (HDAC2, mSin3A, and MeCP2) and revising abnormal synaptic transmission. In contrast, SFN did not affect the protein expression of BDNF and its transcriptional repressor proteins in the medial prefrontal cortex (mPFC) and hippocampus, nor did it reduce depression-like behaviors and abnormal synaptic transmission in *Nrf2* knockout mice. In the mouse model of chronic social defeat stress (CSDS), protein levels of Nrf2 and BDNF in the mPFC and hippocampus were lower than those of control and CSDS-resilient mice. In contrast, the protein levels of BDNF transcriptional repressors in the CSDS-susceptible mice were higher than those of control and CSDS-resilient mice. These data suggest that Nrf2 activation increases the expression of *Bdnf* and decreases the expression of its transcriptional repressors, which result in fast-acting antidepressant-like actions. Furthermore, abnormalities in crosstalk between Nrf2 and BDNF may contribute to the resilience versus susceptibility of mice against CSDS.

## Introduction

The World Health Organization estimates that major depressive disorder (MDD) is the most common psychiatric disorder worldwide, which affects more than 264 million individuals of all ages across the globe. As a result, MDD is a major contributor to the global burden of disease^1^. However, the precise molecular and cellular mechanisms underlying depression remain misunderstood.

Nuclear factor erythroid 2-related factor 2 (Nrf2) is a key transcription factor that regulates antioxidant and anti-inflammatory responses^2–7^. Accumulating evidence suggests a crucial role of Nrf2 in the pathogenesis of depression^8,9^. We reported that Nrf2 activator sulforaphane (SFN) showed antidepressant-like effects in the lipopolysaccharide (LPS)-induced and chronic social defeat stress (CSDS) models of depression by stimulating the expression of brain-derived neurotrophic factor (BDNF)^8,10,11^, and that Nrf2 activators such as TBE-31 and MCE-1 showed antidepressant-like effects in LPS-induced model of depression^12^. Furthermore, we reported that the *Nrf2* knockout (KO) mice showed decreased levels of BDNF and its receptor tropomyosin-receptor-kinase B (TrkB) in the brain, thus resulting in depression-like behaviors^11^. Furthermore, we found that the TrkB agonist 7,8-dihydroxyavone produced antidepressant-like effects in *Nrf2* KO mice^11^. A recent study demonstrated that seasonal changes in the Nrf2 pathway may regulate winter depression-like behaviors^13^. In the learned helplessness (LH) paradigm, the protein levels of Nrf2 and BDNF in the medial prefrontal cortex (mPFC) and hippocampus from LH (susceptible) rats were lower than those of the control and non-LH (resilient) rats^14,15^. Collectively, abnormalities in Nrf2 and BDNF crosstalk in the brain may play a role in causing depression-like phenotypes in rodents.

Multiple lines of evidence support the role of BDNF–TrkB signaling in the pathophysiology of depression and the therapeutic mechanisms of antidepressants and candidates including ketamine and d-serine^16–26^. We reported reduced levels of BDNF in the mPFC and hippocampus of rodents with depression-like phenotypes^15,27–29^. In contrast, we did not find significant changes in BDNF levels between the non-LH (resilient) rats and control rats, thus suggesting that regional differences in BDNF levels in rat brain may promote resilience to inescapable electric stress^30^. Moreover, the overexpression of BDNF in the hippocampus promoted resilience to stress^31^. Transgenic mice overexpressing the full-length TrkB (TrkB.TK^+^) protein, which had an overactive TrkB-PLCγ signaling, exhibited decreased depression-like actions with modified expressions of several plasticity-related genes^32–34^. BDNF heterozygous mice, which exhibit a ~50% decrease in brain levels of BDNF compared with control mice, showed increased vulnerability to developing consequences of stress exposure^17^. These results show that decreased levels of BDNF in the mPFC and hippocampus may contribute to the pathophysiology of depression, whereas activation of the BDNF–TrkB signaling pathway may promote resilience. Taken together, it is important to study the relationship between the Nrf2 and BDNF in rodents with depression-like behaviors. However, the precise molecular mechanisms underlying Nrf2 and BDNF crosstalk in depression are currently unknown.

In the present study, we hypothesized that Nrf2 may regulate the transcription of *Bdnf* by binding to its promoter and causing the dissociation of transcriptional repressors (i.e., HDAC2, mSin3a, and MeCP2) from the promoter. First, we examined whether Nrf2 activation can affect the expression of BDNF and BDNF transcriptional repressor (i.e., HDAC2, mSin3a, and MeCP2). Second, we examined whether Nrf2 regulates *Bdnf* transcription using luciferase assay and chromatin immunoprecipitation (ChIP) assay. Third, we examined the role of Nrf2 and BDNF crosstalk on the depression-like phenotypes in LPS-treated mice and *Nrf2* KO mice. Finally, we examined the role of Nrf2 and BDNF in resilience versus susceptibility in mice after CSDS.

## Materials and methods

### Mice, cell lines, primer information, antibody information, and drug treatment

Male adult C57BL/6 mice (8 weeks old, 20–25 g each, Guangdong Experimental Animal Center), CD1 mice (14 weeks old, 40–45 g each, Guangdong Experimental Animal Center), and male adult *Nrf2* homozygous KO mice (*Nrf2*^−^^/−^) were used in experiments. Age-matched animals from each genotype were randomly allocated to experimental groups. The sample sizes were based on previous experience with the experimental design^10^. Since several batches of mice were tested independently and pooled together for final analyses. Therefore, the group sizes are not exactly the same. The criteria were not pre-established. The animals were housed under controlled temperature and kept on a 12-h light/dark cycle (lights on between 07:00 and 19:00), with ad libitum access to food and water. The protocol was approved by the Jinan University Institutional Animal Care and Use Committee. All experiments were carried out following the Guide for Animal Experimentation of Jinan University. HEK293 or Neuro-2a cells were a gift from Dr. Zhentao Zhang (Department of Neurology, Renmin Hospital of Wuhan University, Wuhan, China) and cultured in high-glucose DMEM supplemented with 10% fetal bovine serum (Excell Bio.) and penicillin (100 units/mL)–streptomycin (100 μg/mL) (all from Hyclone). Cells were incubated at 37 °C in a humidified incubator containing 5% CO_2_. All the cell lines were tested for mycoplasma contamination.

Primer sequences used were for *Nrf2*, *Bdnf*, and *Gapdh* quantitative real-time PCR assay as follows: forward 5ʹ CGAGATATACGCAGGAGAGGTAAGA 3ʹ; reverse 5ʹ GCTCGACAATGTTCTCCAGCTT 3ʹ for *Nrf2*, forward 5ʹ TTGTTTTGTGCCGTTTACCA3ʹ; reverse 5ʹ GGTAAGAGAGCCAGCCACTG 3ʹ for *Bdnf*, and forward, 5ʹ ATGACATCAAGAAGGTGGTG 3ʹ, reverse, 5ʹ CATACCAGGAAATGAGCTTG 3ʹ for *Gapdh*.

The following antibodies were purchased from Abcam: Nrf2 (ab137550), BDNF (ab108319), HDAC2 (ab12169), mSin3A (ab3479), and MeCP2 (ab2828). The beta-actin antibody was purchased from EarthOx.

On the day of injection, fresh solutions were prepared by dissolving drug compounds in sterile endotoxin-free isotonic saline. LPS (0.5 mg/kg; L-4130, serotype 0111: B4, Sigma-Aldrich, St. Louis, MO, USA) was dissolved in physiological saline. SFN (10 mg/kg; MedChemExpress, Shanghai, China) was dissolved in distilled water containing 10% corn oil. LPS (0.5 mg/kg) and SFN (10 mg/kg) were administered intraperitoneally (i.p.) according to previous reports by a researcher blind to the treatment^10,27,28^. d(−)-2-amino-5-phosphonovaleric acid (AP5), 6-cyano-7-nitroquinoxaline-2,3-dione (CNQX), and Bicuculine methiodide (BMI) were purchased from Tocris Bioscience. AP5 (100 μM) and CNQX (20 μM) were used to verify spontaneous excitatory postsynaptic currents (sEPSCs) and BMI (20 μM) was used to verify spontaneous inhibitory postsynaptic currents (sIPSCs)

### CSDS model

CSDS was induced according to previously reported procedures (for details, see Supplementary Information)^35–38^.

### Behavioral tests

Behavioral tests including locomotion, tail suspension test (TST), forced swimming test (FST), and 1% sucrose preference test were performed according to previously reported procedures (for details, see Supplementary Information)^10,27,28^.

### Cell transfection

The mouse plasmid (pcDNA3.1-*Nrf2*) was a gift from Dr. Masayuki Yamamoto (Tohoku University Graduate School of Medicine, Sendai, Japan). The mouse *Bdnf* exons I, II, and IV luciferase plasmids were structured by TsingKe Biological Technology (Wuhan, China). siRNAs were purchased from Santa Cruz. HEK293 or Neuro-2a cells were transfected using Lipofectamine 3000 (Invitrogen) according to the manufacturer’s instructions for cell 48 h.

### Western blotting assay, luciferase assay, ChIP assay, immunofluorescence staining, and electrophysiological recordings

We performed western blot, luciferase assay, ChIP assay, and immunofluorescence staining and obtained electrophysiological recordings for in vitro and/or in vivo experiments (for details, see Supplementary Information).

### Statistical analysis

Data are expressed as the mean ± standard error of the mean (SEM). Data were analyzed using PASW Statistics 20 software. Data were analyzed using Student’s *t*-test or one-way analysis of variance, followed by post hoc Fisher’s least significant difference test. All *p*-values <0.05 were considered statistically significant.

## Results

### Role of Nrf2 in the expression of BDNF transcriptional repressors (MeCP2, HDAC2, and mSin3a)

**Fig. 1:**
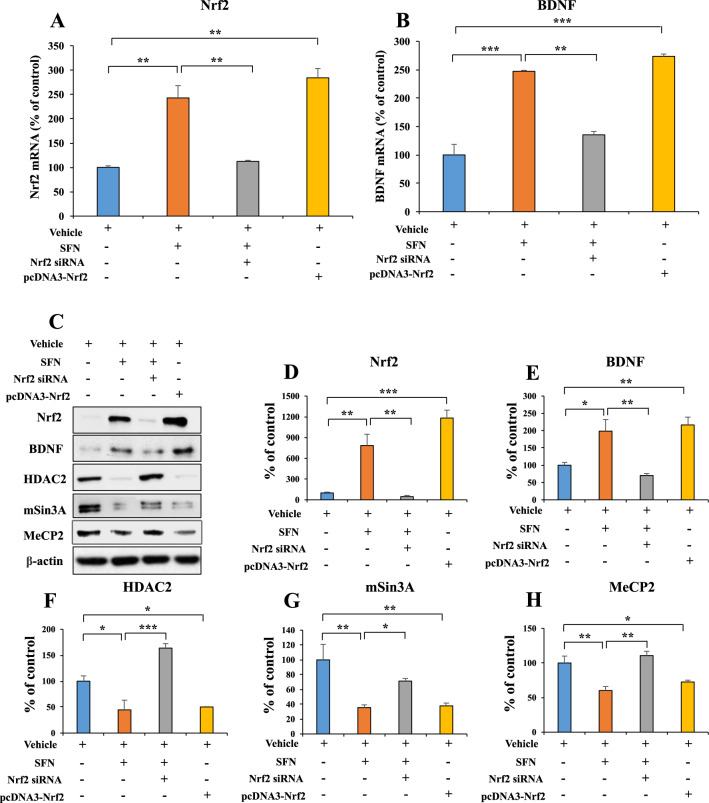
Activation of Nrf2 inhibits BDNF transcriptional repressor (MeCP2, HDAC2, and mSin3a) expression in Neuro-2a cell. **A**, **B** The quantitative real-time PCR for *Nrf2* and *Bdnf* (mean ± SEM, *n* = 3 per group, one-way ANOVA, **p < 0.01 and ***p < 0.01). **C** The representative image for western blot. **D** The quantitative result for Nrf2. **E** The quantitative result for BDNF. **F** The quantitative result for HDAC2. **G** The quantitative result for mSin3A. **H** The quantitative result for MeCP2 (mean ± SEM, *n* = 3 per group, one-way ANOVA, *p < 0.05, **p < 0.01, and ***p < 0.01).

We previously reported that the Nrf2 activator SFN ameliorated the decreased expression of BDNF protein in the brain of mice with a depression-like phenotype^10,11,27^. Hence, we examined to explore whether Nrf2-mediated increases in BDNF affect the expression of *Bdnf* transcriptional repressors (Fig. 1A–H). SFN and/or siRNA-*Nrf2* plasmids for Neuro-2a cells were used. SFN increased the mRNA and protein expression of BDNF and decreased the protein levels of HDAC2, mSin3a, and MeCP2. However, these effects by SFN were blocked by siRNA-*Nrf2*. Moreover, overexpression of Nrf2 in Neuro-2a cells increased the mRNA and protein levels of BDNF while decreasing the protein levels of HDAC2, mSin3a, and MeCP2 (Fig. 1A–H). These results suggest that Nrf2 activation can increase BDNF mRNA and protein expression, whereas Nrf2 activation decreases the expression of HDAC2, mSin3a, and MeCP2 protein.

### Nrf2 mediates *Bdnf* transcription

**Fig. 2:**
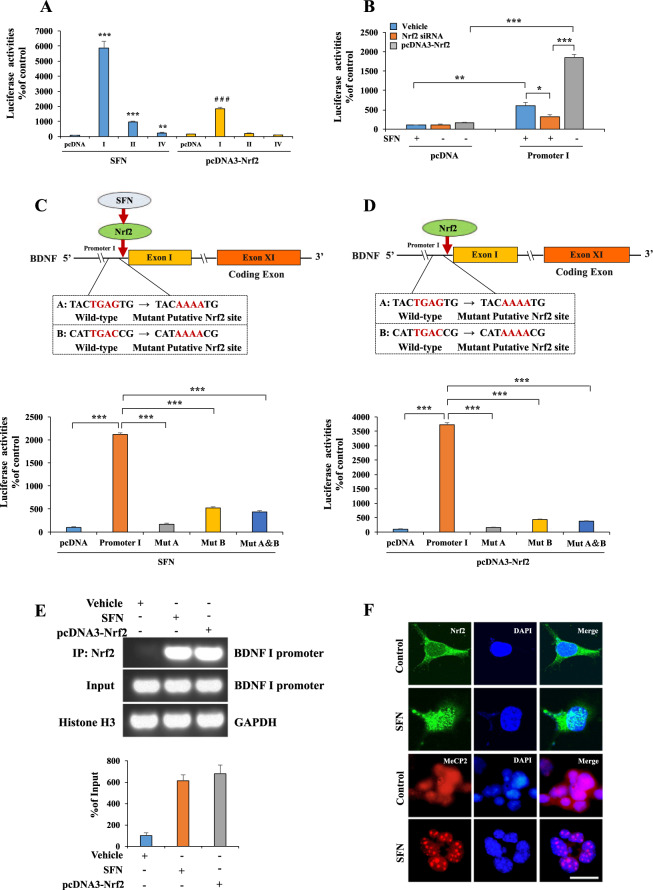
Nrf2 acts as transcription factor for BDNF. **A**–**E** Nrf2 acted as a transcription factor for BDNF promoters. **A**, **B** BDNF exons I, II, and IV luciferase promoters and SFN or Nrf2 or siRNA-Nrf2 plasmids were treated into HEK293 cells (mean ± SEM, *n* = 4 per group, one-way ANOVA, *p < 0.05, **p < 0.01, ***p < 0.001, and ^# # #^*p* < 0.001). **C**, **D** Results obtained using the luciferase plasmids containing mutation (Mut) at the Nrf2 binding motif of BDNF exon I promoter was compared with the wild type of promoters, respectively (mean ± SEM, *n* = 4 per group, one-way ANOVA, *p < 0.05, **p < 0.01, and ***p < 0.001). **E** ChIP-PCR assays demonstrated Nrf2 specifically bound to genomic DNA of BDNF exon I promoter binding motifs. The Nrf2 protein–DNA crosslinking samples were obtained from the HEK293 cells treated with SFN or Nrf2 plasmid or not (control) via co-immunoprecipitating with anti-Nrf2 antibodies. PCR was carried out by using primer pairs at BDNF exon I promoter. PCR assay also included each input sample. The positive control was demonstrated with anti-Histone H3 antibody coupling with GAPDH primers. **F** The immunofluorescence for Nrf2 and MeCP2. The SFN treated for Neuro-2a cell 24 h. The immunofluorescence was performed for Nrf2 and MeCP2. Scale bar 50 μm.

To further determine the mechanism underlying the transcriptional regulation of Nrf2 on *Bdnf*, we analyzed the DNA sequences of the promoter regions in mouse *Bdnf* exons I, II, and IV promoters. We found two potential Nrf2 consensus binding motifs in *Bdnf* exon I promoter based on Nrf2 binding characteristics^39^ (Supplementary Fig. 1). Accordingly, we prepared a series of luciferase-conjugated constructs with the promoters for the *Bdnf* exons I, II, and IV sequences. The luciferase assay using lysates from HEK293 cells treated with SFN or co-transfected with Nrf2 or siRNA-*Nrf2* and the promoter constructs showed that *Bdnf* exon I promoter might be the major binding site for Nrf2 (Fig. 2A, B). The mutation in this motif completely abolished promoter activity (Fig. 2C, D). To further confirm that Nrf2 acts as a transcription factor for *Bdnf*, we carried out a ChIP assay with cells treated with SFN or Nrf2 by using an Nrf2-specific antibody. PCR analysis with genomic DNA immunoprecipitated using the Nrf2 antibody demonstrated that Nrf2 interacted with the *Bdnf* exon I promoter (Fig. 2E). Immunofluorescence staining revealed that SFN treatment caused the redistribution of Nrf2 and MeCP2 for the nucleus. We found that more Nrf2 within the nucleus and the more diffused nuclear staining pattern of MeCP2 in vehicle-treated cells became more punctate upon SFN treatment (Fig. 2F). These results suggest that Nrf2 functions as a transcription factor for *Bdnf*.

### The roles of Nrf2 in depression-like behaviors and the expression of BDNF after LPS administration

**Fig. 3:**
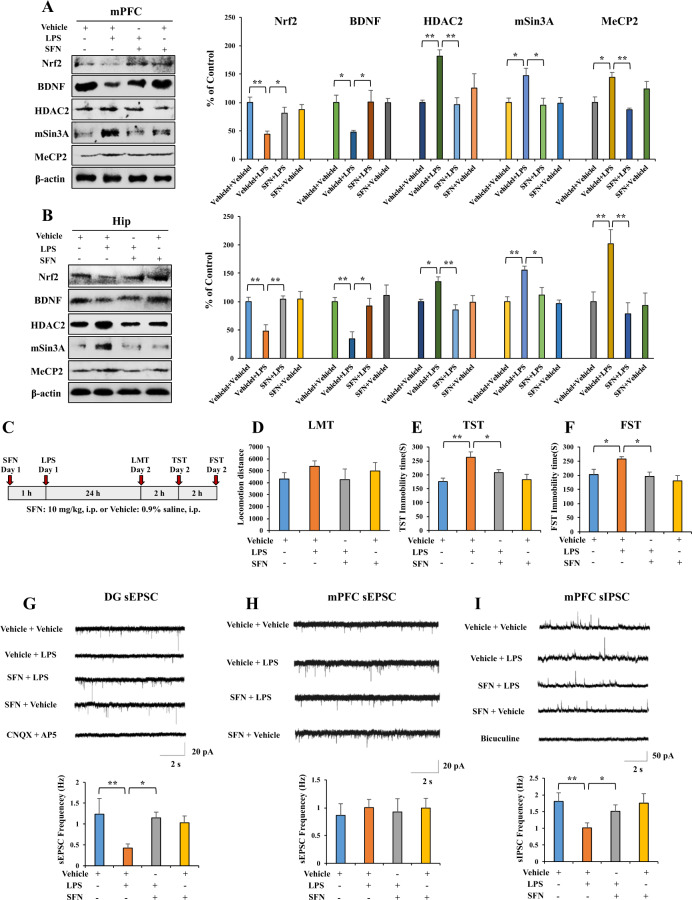
SFN shows beneficial effects for depression-like behavior by altering Nrf2, BDNF, HDAC2, mSin3a, MeCP2 expression, and attenuating the decrease of frequency of sEPSC and sIPSC in hippocampus and mPFC neurons. **A**, **B** Western blot analysis for Nrf2, BDNF, and MeCP2 in mPFC and hippocampus (mean ± SEM, *n* = 4 per group, one-way ANOVA, *p < 0.05 and **p < 0.01). **C** The schedule of behavior test and treatment. **D** LMT: locomotion test, **E** TST: tail-suspension test, **F** FST: forced swimming test (mean ± SEM, *n* = 7 or 8 per group, one-way ANOVA, *p < 0.05, **p < 0.01). **G** Up: representative traces of sEPSC in hippocampal DG neurons. Scale bars represent 2 s, 20 pA. Down: histograms of sEPSC frequency in hippocampal DG neurons (mean ± SEM, *n* = 7–9 neurons of three mice per group, one-way ANOVA, *p < 0.05, **p < 0.01). **H** Up: representative traces of sEPSC in mPFC neurons. Scale bars represent 2 s, 20 pA. Down: histograms of sEPSC frequency in mPFC neurons (mean ± SEM, *n* = 8–10 neurons of three mice per group). **I** Up: representative traces of sIPSC in mPFC neurons. Scale bars represent 2 s, 50 pA. Down: histograms of sIPSC frequency in mPFC neurons (mean ± SEM, *n* = 8–10 neurons of three mice per group, one-way ANOVA, *p < 0.05, **p < 0.01).

We examined whether Nrf2 plays a role in LPS-induced depression-like behaviors by elevating BDNF expression and inhibiting BDNF transcriptional repressor expression. First, we found that LPS (0.5 mg/kg, i.p.) significantly decreased the levels of Nrf2 and BDNF and increased the levels of HDAC2, mSin3a, and MeCP2 protein in the mPFC and hippocampus (Fig. 3A, B). SFN (10 mg/kg, i.p.) significantly attenuated the reduction in Nrf2 and BDNF protein expression observed after LPS administration and increased the levels of MeCP2, HDAC2, and mSin3a protein in the mPFC and hippocampus (Fig. 3A, B). Next, we used immunofluorescence to visualize Nrf2 and MeCP2 in the selected brain regions. LPS significantly decreased the Nrf2 fluorescence intensity, whereas LPS significantly increased the MeCP2 fluorescence intensity in the mPFC and dentate gyrus (DG) of the hippocampus. SFN significantly attenuated the reduction in Nrf2 fluorescence intensity and increased the MeCP2 fluorescence intensity in the mPFC and DG of the hippocampus after a single dose of LPS (Supplementary Fig. 2A, B).

Moreover, we performed the behavioral tests to examine the antidepressant-like effects of SFN in LPS-induced depression-like behaviors (Fig. 3C). There were no changes in locomotion observed among the four groups (Fig. 3D). SFN significantly attenuated the increased immobility time of TST and FST observed after LPS administration (Fig. 3E, F). However, SFN alone did not affect the immobility time during the TST and FST in control mice (Fig. 3E, F).

Previous studies showed that depression-like behaviors are associated with impaired synaptic transmission in the hippocampus and mPFC^40–42^, and BDNF is an important regulator of synaptic transmission^43^. Therefore, we obtained whole-cell patch-clamp recordings in acute hippocampus slices and recorded sEPSCs. LPS treatment significantly decreased the frequency of sEPSC without affecting the amplitude in neurons of the hippocampal DG (Fig. 3G and Supplementary Fig. 3A). Furthermore, SFN significantly attenuated the decrease in the sEPSC frequency in hippocampal DG neurons after LPS administration (Fig. 3G). However, SFN alone did not affect the frequency or amplitude of sEPSCs in hippocampal DG neurons (Supplementary Fig. 3A). sEPSC could be completely blocked by the AMPA and NMDA receptor antagonists AP5 and CNQX. We recorded sEPSCs of mPFC neurons to further determine whether LPS administration can affect glutamatergic synaptic transmission in mPFC neurons. However, LPS and SFN administration did not affect the frequency and amplitude of sEPSCs in mPFC neurons (Fig. 3H and Supplementary Fig. 3B). Because disrupted GABA neurotransmission in the mPFC contributes to the neurobiology of depression^44^, we tested whether GABAergic transmission was altered after LPS injection. Surprisingly, we found that LPS treatment significantly decreased the frequency of sIPSCs without affecting the amplitude of mPFC neurons. Furthermore, this change could be reversed by SFN treatment (Fig. 3I and Supplementary Fig. 3C). SFN alone did not affect the frequency or amplitude of sIPSCs in mPFC neurons (Fig. 3I and Supplementary Fig. 3C). sIPSCs were inhibited by bicuculline methiodide (BMI), a GABA_A_ receptor antagonist.

### Role of Nrf2 in depression-like phenotypes and changes in BDNF, HDAC2, mSin3a, and MeCP2 expression

**Fig. 4:**
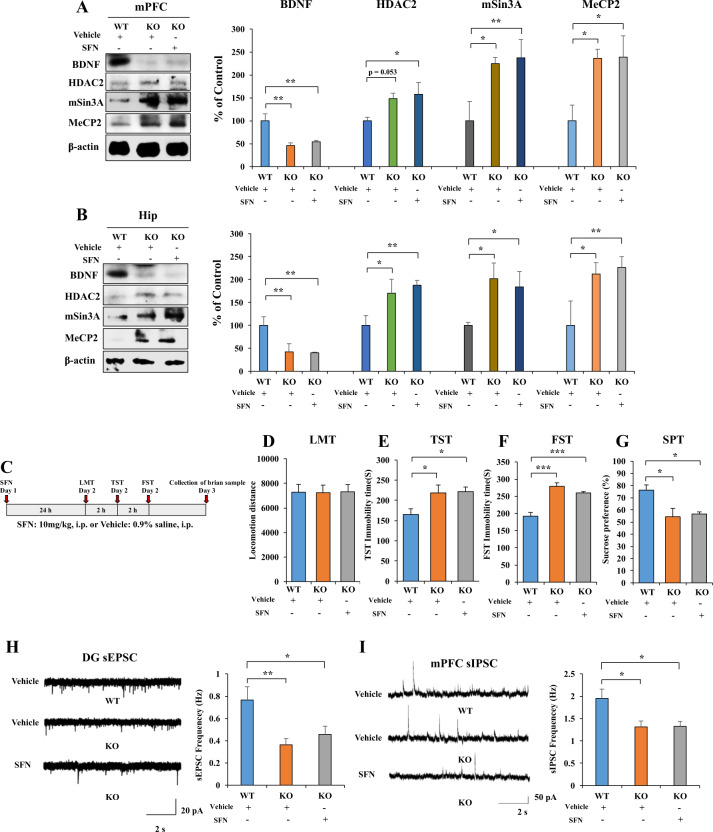
Nrf2 KO mice show depression-like behavior, alter BDNF, HDAC2, mSin3a, and MeCP2 expression and frequency of sEPSC and sIPSC in hippocampus and mPFC neurons. **A**, **B** The western blot analysis for mPFC and hippocampus. The western blot was performed for Nrf2, BDNF, HDAC2, mSin3A, and MeCP2 in mPFC and hippocampus (mean ± SEM, *n* = 4 per group, one-way ANOVA, *p < 0.05, **p < 0.01). **C** The behavior test for *Nrf2* KO mice after the SFN treatment. **D** LMT: locomotion test, **E** TST: tail-suspension test, **F** FST: forced swimming test, **G** SPT: sucrose preference test (mean ± SEM, *n* = 8 per group, one-way ANOVA, *p < 0.05, **p < 0.01). **H** Left: representative traces of sEPSC in hippocampal DG neurons. Scale bars represent 2 s, 20 pA. Right: histograms of sEPSC frequency in hippocampal DG neurons (mean ± SEM, *n* = 10–11 neurons of three mice per group, one-way ANOVA, *p < 0.05, **p < 0.01). **I** Left: representative traces of sIPSC in mPFC neurons. Scale bars represent 2 s, 50 pA. Right: histograms of sIPSC frequency in mPFC neurons (mean ± SEM, *n* = 9–10 neurons of three mice per group, one-way ANOVA, *p < 0.05).

We used *Nrf2* KO mice with a depression-like phenotype to confirm the role of Nrf2 and BDNF crosstalk in the pathogenesis of depression^11^. Using western blot, we analyzed BDNF, HDAC2, mSin3A, and MeCP2 protein expression in the selected brain regions from wild type (WT) mice and *Nrf2* KO mice. Protein levels of BDNF in the mPFC and hippocampus of *Nrf2* KO mice were significantly lower than those of WT mice. Furthermore, protein levels of HDAC2, mSin3A, and MeCP2 in the mPFC and hippocampus were significantly higher than those of WT mice; the change in HDAC2 expression in the mPFC was not statistically significant (Fig. 4A, B). Treatment of SFN did not change the expression of these proteins in *Nrf2* KO mice (Fig. 4A, B). These data suggest the role of Nrf2 and BDNF crosstalk in the depression-like phenotypes of *Nrf2* KO mice.

*Nrf2* KO mice showed depression-like behaviors, which was consistent with our previous report^11^. In contrast, SFN (10 mg/kg) did not show antidepressant-like effects in *Nrf2* KO mice (Fig. 4C–G). There were no differences in locomotion among the three groups (Fig. 4D). Regarding the TST and FST, the increased immobility times of *Nrf2* KO mice were not improved after SFN (10 mg/kg) administration (Fig. 4E, F). Furthermore, the decreased sucrose preference of *Nrf2* KO mice was not improved after the administration of SFN (Fig. 4G). These data suggest the role of Nrf2 in the antidepressant-like effects of SFN.

Moreover, we also performed whole-cell patch-clamp recordings in acute hippocampus and mPFC slices and recorded sEPSCs and sIPSCs for *Nrf2* KO mice. In *Nrf2* KO mice, the frequency of sEPSC is significantly decreased without affecting the amplitude in neurons of the hippocampal DG (Fig. 4H and Supplementary Fig. 4A). SFN (10 mg/kg) did not alter the frequency of sEPSC in *Nrf2* KO mice (Fig. 4H).

LPS significantly decreased the frequency of sIPSC in neurons of mPFC, and SFN (10 mg/kg) significantly attenuated the decreased frequency of sIPSC in neurons of mPFC (Fig. 3I). Therefore, we examined the frequency and amplitude of sIPSC in neurons of mPFC for *Nrf2* KO mice. In *Nrf2* KO mice, the frequency of sIPSC is significantly decreased without affecting the amplitude in neurons of the mPFC (Fig. 4I and Supplementary Fig. 4B). SFN (10 mg/kg) did not alter the frequency and amplitude of sIPSC in *Nrf2* KO mice (Fig. 4I and Supplementary Fig. 4B).

### Role of Nrf2, BDNF, and BDNF transcriptional repressor proteins in the CSDS model

**Fig. 5:**
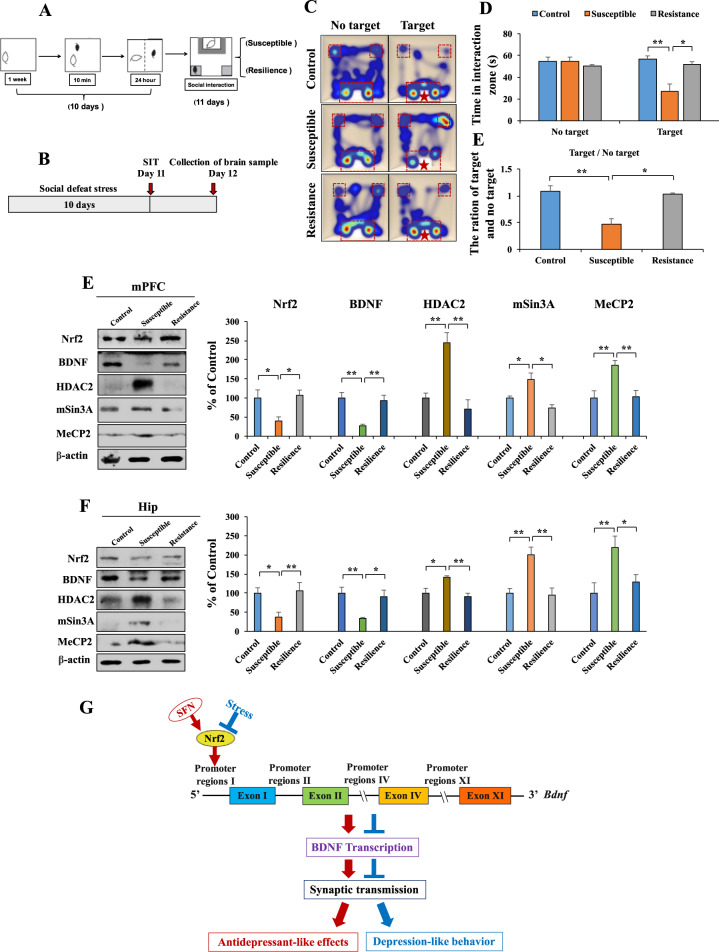
Resistance elevates Nrf2, BDNF expression and inhibits HDAC2, mSin3a, and MeCP2 expression. **A**, **B** Schematic of social defeat stress model and the schedule of treatment. **C** Thermal imaging of mice trajectories. **D** The social interaction test for no target and target time (mean ± SEM, *n* = 4–6 per group, one-way ANOVA, *p < 0.05, **p < 0.01). **E** The ration for target and no target time (mean ± SEM, *n* = 4–6 per group, one-way ANOVA, *p < 0.05, **p < 0.01). **E**, **F** The western blot analysis for mPFC and hippocampus. The western blot was performed for Nrf2, BDNF, HDAC2, mSin3A, and MeCP2 in mPFC and hippocampus (mean ± SEM, *n* = 4 per group, one-way ANOVA, *p < 0.05, **p < 0.01). **G** The working model of Nrf2-induced BDNF transcription in the model of depression. Stress inhibits Nrf2 expression, which leads to inhibit BDNF transcriptional and abnormal synaptic transmission, causing depression-like behaviors in mice. SFN induces BDNF transcription by activating Nrf2 and could correct the abnormal synaptic transmission, resulting in antidepressant-like effects.

Previously, we reported that the protein expression of Nrf2 and BDNF in the mPFC and hippocampus of LH (susceptible) rats was lower than that of control and non-LH (resilient) rats^14,15^. Here we used the CSDS model of depression. Using the social interaction test, we divided the CSDS-susceptible and CSDS-resilient mice (Fig. 5A–E). We observed decreased expression of Nrf2 and BDNF in the mPFC and hippocampus from CSDS-susceptible mice, but not CSDS-resilient mice, compared with that from the control (no CSDS) mice (Fig. 5E, F). In contrast, we observed increased expression of HDAC2, mSin3A, and MeCP2 in the mPFC and hippocampus from CSDS-susceptible mice, but not CSDS-resilient mice, compared with that from the control (no CSDS) mice (Fig. 5E, F). There were no differences in change of these transcriptional repressor proteins between CSDS-resilient mice and control (no CSDS) mice (Fig. 5E, F).

## Discussion

The major findings of this study are as follows. First, the activation of Nrf2 by SFN or *Nrf2* gene transfection increased the expression of *Bdnf* mRNA and protein while decreasing the expression of its transcriptional repressors (HDAC2, mSin3a, and MeCP2) in Neuro-2a cells. These effects were blocked by siRNA-Nrf2, suggesting a role for Nrf2. Second, using the luciferase and ChIP assays, we found that Nrf2 binds to the *Bdnf* exon I promoter, indicating that Nrf2 is a transcription factor for *Bdnf*. Third, we found decreased expression of Nrf2 and BDNF protein levels in the mPFC and hippocampus from LPS-treated mice, whereas the protein expression of BDNF transcriptional repressors (HDAC2, mSin3a, and MeCP2) was increased in LPS-treated mice. Fourth, SFN showed antidepressant-like effects by normalizing the decreased levels of Nrf2 and BDNF protein; increased levels of HDAC2, mSin3a, and MeCP2 protein; and abnormal synaptic transmission observed in LPS-treated mice. Fifth, SFN did not show antidepressant-like effects nor ameliorate the altered expression of BDNF, HDAC2, mSin3A, and McCP2; and abnormal synaptic transmission observed in the mPFC and hippocampus of *Nrf2* KO mice. Finally, we found that decreased expression of Nrf2 and BDNF as well as increased expression of HDAC2, mSin3A, and MeCP2 in the mPFC and hippocampus might confer stress susceptibility in mice after CSDS. The downregulation of BDNF caused by reduced Nrf2 activity may play a key role in depression-like phenotypes in rodents.

It is reported that Nrf2 binds to the antioxidant responsive element, which is located in the promoter region of genes encoding many detoxifying or antioxidant enzymes and related stress-responsive proteins^2–4,6^. We previously reported that pretreatment with SFN or dietary intake of 0.1% glucoraphanin (a precursor for SFN) significantly attenuated depression-like phenotypes and altered BDNF expression in the mPFC and hippocampus of mice with depression-like phenotypes^10,11^. Using the luciferase and ChIP assays, we confirmed that Nrf2 binds to the *Bdnf* exon I promoter at two binding sites. Using immunofluorescence, we observed the SFN-induced redistribution of Nrf2 and MeCP2 for the nucleus. We found that more Nrf2 within the nucleus and the more diffused nuclear staining pattern of MeCP2 in vehicle-treated cells became more punctate upon SFN treatment. These data suggest that Nrf2 functions as a transcription factor for *Bdnf*.

Nrf2 can interact with its principal negative regulator, the E3 ligase adapter Kelch-like ECH-associated protein (Keap1)^2–7^. We reported decreased expression of Nrf2 and Keap1 in the parietal cortex from MDD patients compared with that from the control group, which indicates that decreased Keap1–Nrf2 signaling may play a crucial role in the development of depression^14^. We also reported alterations in the expression of BDNF, BDNF pro-peptide, and their precursor (proBDNF) in the parietal cortex of patients with major psychiatric disorders, including MDD^45^. Thus, abnormalities in the processing of BDNF and BDNF pro-peptide from proBDNF in the parietal cortex may contribute to the pathogenesis of major psychiatric disorders. Furthermore, Bouvier et al.^46^ showed that the reduced levels of BDNF in mice with depression-like behavior could prevent Nrf2 translocation and the activation of detoxifying/antioxidant enzymes, which ultimately sustained oxidative stress. Furthermore, it is suggested that BDNF plays a key role as an inducer of neuronal antioxidant responses by BDNF-induced Nrf2 nuclear translocation^47^. A recent study demonstrated the role of BDNF–TrkB signaling in the antidepressant-like effects of (*R*)-ketamine in *Nrf2* KO mice^48^. Therefore, it is likely that the communication between Keap1–Nrf2 signaling and BDNF–TrkB signaling in the brain might play a crucial role in depression (Fig. 5G).

Rodents with depression-like phenotypes show impaired glutamatergic signaling in the hippocampus and GABAergic transmission in the mPFC^40,49^. LPS administration impaired the sEPSC frequency of hippocampal neurons and sIPSC frequency of mPFC neurons. Also, SFN reversed impairments in the glutamatergic transmission of hippocampal neurons and GABAergic transmission in mPFC neurons among LPS-treated mice. We found that sEPSC frequency of hippocampal neurons and sIPSC frequency of mPFC neurons of *Nrf2* KO mice are abnormal, and that SFN could not reverse impairments in the glutamatergic transmission of hippocampal neurons and GABAergic transmission in mPFC neurons of *Nrf2* KO mice. Considering the role of Nrf2 as the transcription factor for *Bdnf*, we propose that the activation of Nrf2 may ameliorate LPS-induced impairments in the glutamatergic transmission of hippocampal neurons and GABAergic transmission of mPFC neurons through BDNF activation. Previous studies showed that the deletion of *Bdnf* could decrease the frequency of sEPSCs in the hippocampus^50^, and *Bdnf* promotor IV mutant mice exhibited significant deficits in GABAergic transmission in the mPFC^51^. Collectively, LPS-induced suppression of Nrf2 expression and subsequent inhibition of *Bdnf* transcription may contribute to depression-like phenotypes and abnormal synaptic transmission in the mice with depression-like phenotype. However, further studies are needed.

Multiple lines of evidence support that resilience is implicated in the development of several psychiatric disorders^52,53^. However, the detailed molecular mechanisms underlying resilience against MDD remain unknown. We previously showed that alterations in the levels of BDNF, BDNF pro-peptide, and pro-BDNF in the mPFC and hippocampus confer stress resilience in the rat LH model^30^. Using the same samples, we reported that the protein levels of Keap1 and Nrf2 in the mPFC and DG of hippocampi from LH (susceptible) rats were lower than those in the control and non-LH (resilience) rats. Therefore, Keap1–Nrf2 signaling may play a role in stress resilience^14^. It is also reported that activation of Nrf2 translocation decreased vulnerability to depression^46^. A recent study demonstrated that decreased Keap1–Nrf2 signaling in the mPFC, hippocampus, and muscle might increase susceptibility to anhedonia after spared nerve injury (SNI) surgery. Furthermore, SFN exerts beneficial effects in SNI rats by attenuating decreased Keap1–Nrf2 signaling^54^. Decreased Nrf2 expression might decrease BDNF expression in the mPFC and hippocampus, resulting in a depression-like phenotype. We found that CSDS-resilient mice showed increased levels of Nrf2 and decreased expression of HDAC2, mSin3a, and MeCP2 in the mPFC and hippocampus compared with CSDS-susceptible mice. Moreover, based on our in vitro studies, Nrf2 binding to the *Bdnf* exon I promoter results in *Bdnf* transcription. Therefore, the inhibition of Nrf2-induced *Bdnf* transcription may contribute to CSDS-induced depression-like behaviors, and activation of Nrf2-induced *Bdnf* transcription may confer stress resilience (Fig. 5G).

In conclusion, Nrf2 regulates the transcription of *Bdnf* by binding to its exon I promoter. Furthermore, the inhibition of Nrf2-induced *Bdnf* transcription may play a role in the pathophysiology of depression. Also, the activation of Nrf2-induced *Bdnf* transcription promoted antidepressant-like effects. Finally, alterations in the crosstalk between Nrf2 and BDNF may contribute to resilience versus susceptibility after stress.

## Supplementary information

Supplemental information
