# Supplementary material for: Activation of BDNF by transcription factor Nrf2 contributes to antidepressant-like actions in rodents
Source: Transl Psychiatry. 2021 Feb 24;11:140. doi: 10.1038/s41398-021-01261-6 (PMC7904924; doi:10.1038/s41398-021-01261-6)
Supplement: Supplementary file 1 — Supplemental information [file 41398_2021_1261_MOESM1_ESM.pdf]

Supplemental Information

Materials and methods

Chronic social defeat stress (CSDS)

For the CSDS depression model, the C57BL/6 mice were exposed to differently CD1 mice for 10 min total of 10 days. The CD1 mouse and C57BL/6 mice were housed in the half cage that separated by using a perforated Plexiglas divider, which can allow visual, olfactory, and auditory contact in the 24 hours after the social defeat session. After finish the last session of social defeat, the C57BL/6 mice were raised separately. The social interaction test was performed to examine the mice that were susceptible and unsusceptible to social defeat stress.

For the social interaction test, an open box (42 × 42 cm) was used, which has an interaction zone including a mesh-plastic target box (10 × 4.5 cm) and two opposing corner zones. This test is divided into two parts (no social target and social target). For the no social target, the test mouse was placed into an open field arena for 2.5 min with no social target (no CD1 mouse) in the mesh-plastic target box. After no social target test, the mouse was placed into an open field arena again in the second 2.5 min with a social target (a novel CD1 mouse) in the mesh-plastic target box. The residence time in the interaction zone was counted by using the stopwatch, the time of ratio for social target, and no social target was calculated. About 70% of mice were susceptible after social defeat stress [1-4].

Behavioral tests

Behavioral tests including locomotion, tail suspension test (TST), forced swimming test (FST), and 1% sucrose preference test (SPT). Locomotion: the locomotor activities of mice were analyzed by using Ethovision XT 14.0 software (Noldus). The cumulative exercise was recorded in 60 minutes. TST: The mice were hung individually by using tape for a hook. The immobility time was recorded when the mice completely motionless in 10 minutes. FST: The mice were placed individually in a cylinder (diameter: 23 cm; height: 31 cm) containing 15 cm of water, maintained at 23 ± 1 °C.

Mice were monitored using a video tracking system (Ethovision XT 14.0) for 6 minutes. SPT: The mice were habituated to a 1% sucrose solution for 48 h before the test day. And then the mice were deprived of water and food for 4 h, followed by a preference test with water and 1% sucrose for 1 h. The bottles containing water and sucrose were weighed before and at the end of this period and the sucrose preference (%) was determined [5-7].

Quantitative real-time PCR assay

Levels of *Nrf2* and *Bdnf* mRNA were analyzed by quantitative real-time PCR. RNA was extracted by using Eastep® Super Kit (Promega), and then reverse transcription was performed with GoScript™ Reverse Transcriptase Mix, Oligo (dT) (Promega). All real-time PCR reactions were performed by using the 788BR05175 Real-Time PCR System and ChamQ™ SYBR® qPCR Master Mix Kit (Vazyme). The target gene expression was calculated as the $2^{-\Delta\Delta Ct}$ method. Forty cycles of PCR amplification were performed as follows: denature at 95°C for 30 s, annealing at 55°C for 30 s, and extend for 30 s at 72°C.

Western blotting assay

Cells or brain samples were lysed in RIPA buffer (20 mM pH 7.5 Tris-HCl, 150 mM NaCl, 1 mM Na_2EDTA, 1 mM EGTA, 1% Triton, 2.5 mM sodium pyrophosphate, 1 mM beta-glycerophosphate, 1 mM Na_3VO_4, 1 μg/ml leupeptin, 1 mM phenylmethylsulfonyl fluoride). The concentrations of total proteins were examined by Bradford assay. 30 μg Proteins were resolved on 7.5%, 10%, or 15% polyacrylamide gels, according to each marker's molecular weight, and then transferred to polyvinylidenedifluoride (PVDF) membrane. For the immunodetection, the blots were blocked with 2% BSA plus 5% nonfat dry milk in TBST (TBS + 0.1% Tween-20) for 1 h at room temperature (RT) and then incubated with primary antibodies (The concentration is selected by the manufacturer's instructions) overnight at 4°C. The next day, the blot was washed three times in TBST and incubated with a horseradish peroxidase-conjugated anti-rabbit antibody (1:5000) or anti-mouse antibody (1:5000) for 1hour, at RT. After the 3 time washes with TBST, the bands were detected by using enhanced chemiluminescence (ECL) detection reagents (GE Healthcare) and exposed

to Tanon-5200CE imaging system (Tanon, Shanghai, China). The quantification was carried out with ImageJ software.

Luciferase assay

Cells in 6-wells plates were transfected with BDNF exon I, II, and IV luciferase reporter together, pRL-TK Renilla luciferase plasmid (Promega), and different kinds of plasmids or siRNA. Following transfection for 48 h, the cells were collected and subjected to the dual-luciferase reporter assay kit (Promega) according to the manual.

Chromatin immunoprecipitation (ChIP) assay

After transfection or treatment with certain plasmids or drugs, the cells were subjected to the ChIP assay according to the procedures described in the manual of the SimpleChIP® Enzymatic Chromatin IP Kit (Cell signaling). For the Nrf2 antibody, 7.5 μg of Nrf2 antibody (Abcam) was added to the homogenate for the sample. For the PCR analysis. Specific primers were for the amplification of the promoter region of 150 bp 5' of BDNF exon I, which contains a putative Nrf2 binding site. The primer sequences were: forward 5'TGATCATCACTCACGACCACG 3'; reverse 5'CAGCCTCTCTGAGCCAGTTACG3' based on previously published results [8]. The 35 cycle PCR was performed as follows: denature at 95°C for 30 s, annealing at 58°C for 30 s and extend for 30 s at 72°C, and then the PCR sample was resolved on a 2% agarose gel and sequenced.

Immunofluorescence staining

After LPS administration 24 hours, mice were anesthetized with sodium pentobarbital and perfused transcardially with 10 ml of isotonic saline, followed by 40 ml of ice-cold 4% paraformaldehyde in 0.1-M phosphate buffer (pH 7.4). After the perfused the brain samples were collected and postfixed overnight at 4°C. On the next day, 50-μm thick serial coronal sections of brain tissue were cut in ice-cold, 0.01-M phosphate-buffered saline (pH 7.5), using a vibrating blade microtome (VT1000S, Leica Microsystems AG,

Wetzlar, Germany). Brain sections were identified according to the previously reported [7]. For the immunofluorescence staining, the slides with cells were fixed by 4% paraformaldehyde. And then the slides with cells or mice brain sections were incubated with 3% hydrogen peroxide at room temperature for 10 minutes followed by blocking and incubation with primary antibodies for 48 h at 4°C. On the third day, the slides with cells or brian sections were incubated with an Alexa Fluor 488 or 568 conjugated isotype-specific secondary antibody for 1 h at room temperature. Images were then collected with an Olympus confocal microscope. The fluorescence intensity was quantified using Image J.

Electrophysiological recordings

For acute slice preparation, all experiments were performed as previously reported [9, 10]. After LPS or SFN administration twenty-four hours, mice were deeply anesthetized with isoflurane and then decapitated, mice brains were quickly and carefully removed and subsequently transferred for ice-cold oxygenated ACSF containing 120 mM NaCl, 2.5 mM KCl, 1.2 mM NaH_2PO_4, 2.0 mM $CaCl_2$, 2.0 mM $MgSO_4$, 26 mM $NaHCO_3$, and 10 mM glucose. mPFC and hippocampus slices (300 mm) were cut by using a VT-1200S vibratome (Leica, Germany) and placed in a storage chamber containing ACSF for a 30-min recovery period at 34°C and then transferred to a room temperature condition (25 ± 1°C) for an additional 2-8 h. All solutions were saturated with 95% O_2/5% CO_2 (vol/vol). To record spontaneous excitatory postsynaptic currents (sEPSC, -70mV hold), glass pipettes (3-6 MΩ) were filled with a solution containing 130 mM potassium gluconate, 20 mM KCl, 10 mM HEPES buffer, 4 mM Mg-ATP, 0.3 mM Na-GTP, 10 mM disodium phosphocreatine and 0.2 mM EGTA, pH 7.2 with KOH, 290 mOsm. sEPSC was verified by adding AP5 (100μM) and CNQX (20μM). To assess spontaneous inhibitory postsynaptic currents (sIPSC, 0mV hold), glass pipettes (3–6 MΩ) were filled with a Cs^+-based peptide solution containing 130 mM CsMeSO4, 10 mM NaCl, 10 mM EGTA. 4 mM Mg-ATP. 0.3 mM Na-GTP, 10 mM HEPES, pH 7.4 with CsOH, 290 mOsm. sIPSC was verified by adding $GABA_A$ receptor antagonist BMI (20μM). A fixed length of traces (2 minutes)

was analyzed for frequency and amplitude distributions of sEPSC or sIPSC using the MiniAnalysis program.

Supplemental figures

BDNF I Promoter

3540 - Nrf2 binding site A

ACTAGTAC TGAGTG CTGGTGCAGGCGACCTCTGGGAATCAGTACCACCCCCTCCCCGTGCTTGAA
AA GATTCTTCAAAGGAAAA ATGGTTCCCTCAGTGTCACTCAACCAGTGCAT TGACCG AGTCTCTC
CGAC -3673

Nrf2 binding site B

Mutation A
ACTAGTAC AAAATG CTGGTGCAGGCGACCTCTGGGAATCAGTACCACCCCCTCCCCGTGCTTGAAAA

Mutation B
GATTCTTCAAAGGAAAAATGGTTCCCTCAGTGTCACTCAACCAGTGCAT AAAACG AGTCTCTCCGAC

Supplemental figure 1. The BDNF exon I promoter sequence and the experiment design for Nrf2 binding site mutation

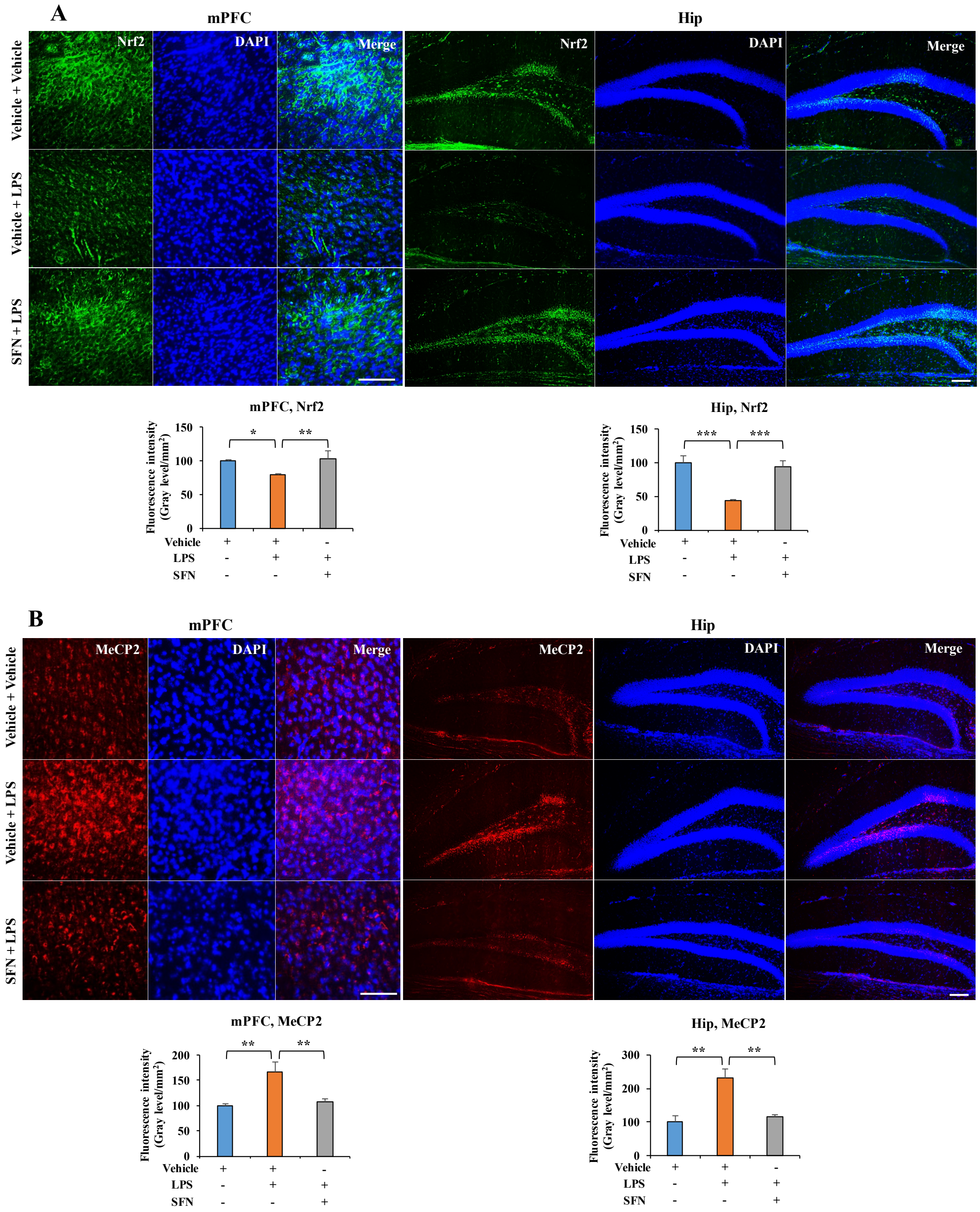

Supplemental figure 2. The immunofluorescence analysis for Nrf2 and MeCP2

A and B: The immunofluorescence analysis for Nrf2 and MeCP2 in mPFC and hippocampus of depression-like behavior mice (Mean ± SEM, n = 4 per group, one-way ANOVA, *$p < 0.05$, **$p < 0.01$ and ***$p < 0.01$). Scar bar = 50 μm.

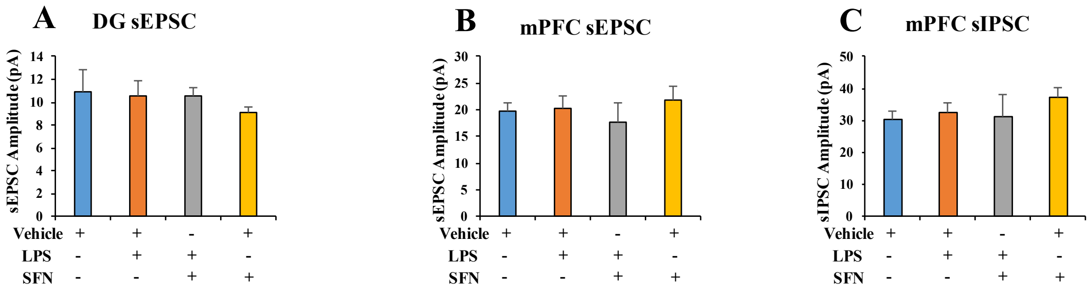

Supplemental figure 3. The electrophysiological recordings for hippocampus and mPFC neurons in LPS-treated mice with depression-like phenotypes

A and B: The sEPSC Amplitude for hippocampus DG neurons and mPFC neurons (Mean ± SEM, n = 7-9 neurons of 3 mice per group for hippocampus DG neurons, and n = 8-10 neurons of 3 mice per group for mPFC, one-way ANOVA). **C**: The sIPSC Amplitude for mPFC neurons (Mean ± SEM, n = 8-10 neurons of 3 mice per group for mPFC, one-way ANOVA).

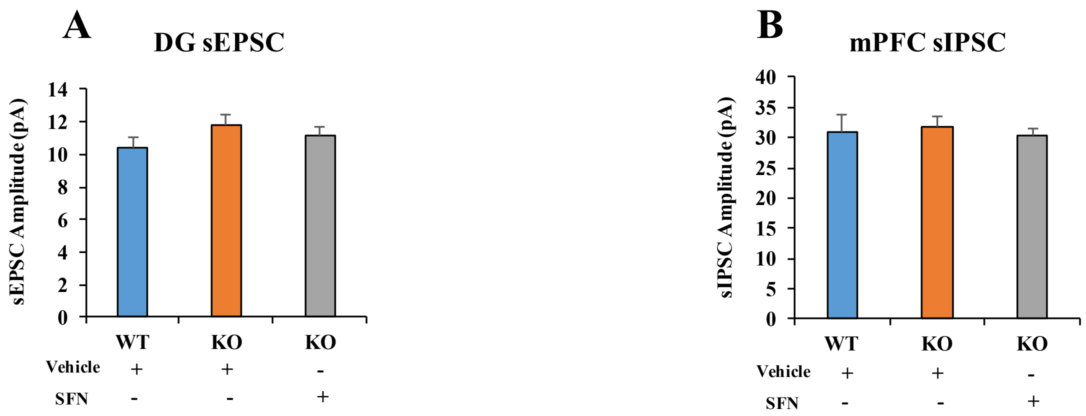

Supplemental figure 4. The electrophysiological recordings for hippocampus and mPFC neurons in *Nrf2* KO mice

A and B: The sEPSC and sIPSC Amplitude for hippocampus DG neurons and mPFC neurons (Mean ± SEM, n = 10-11 neurons of 3 mice per group for hippocampus DG neurons, and n = 9-10 neurons of 3 mice per group for mPFC, one-way ANOVA).

Reference

1. Berton, O., *et al.* Essential role of BDNF in the mesolimbic dopamine pathway in social defeat stress. *Science* **311**, 864-868 (2006).
2. Golden, S.A., Covington, H.E., 3rd, Berton, O. & Russo, S.J. A standardized protocol for repeated social defeat stress in mice. *Nat. Protocols* **6**, 1183-1191 (2011).
3. Tsankova, N.M., Berton, O., Renthal, W., Kumar, A., Neve, R.L. & Nestler, E.J. Sustained hippocampal chromatin regulation in a mouse model of depression and antidepressant action. *Nat. Neurosci* **9**, 519-525 (2006).
4. Zhao, T., *et al.* Effects of chronic social defeat stress on behavior and choline acetyltransferase, 78-kDa glucose-regulated protein, and CCAAT/enhancer-binding protein (C/EBP) homologous protein in adult mice. *Psychopharmacology (Berl)* **228**, 217-230 (2013).
5. Zhang, J.C., *et al.* Antidepressant effects of TrkB ligands on depression-like behavior and dendritic changes in mice after inflammation. *Int. J. Neuropsychopharmacol.* **18**, pyu077 (2014).
6. Zhang, J.C., *et al.* Comparison of ketamine, 7,8-dihydroxyflavone, and ANA-12 antidepressant effects in the social defeat stress model of depression. *Psychopharmacology (Berl)* **232**, 4325-4335 (2015).
7. Zhang, J.C., *et al.* Prophylactic effects of sulforaphane on depression-like behavior and dendritic changes in mice after inflammation. *J. Nutr. Biochem.* **39,** 134-144 (2017).
8. Kim, J., *et al.* Sulforaphane epigenetically enhances neuronal BDNF expression and TrkB signaling pathways. *Mol. Nutr. Food Re.s* 2017; **61**(2). doi: 10.1002/mnfr.201600194.
9. Huang, L., *et al.* A visual circuit related to habenula underlies the antidepressive effects of light therapy. *Neuron* **102**, 128-142 e128 (2019).
10. Lin, S., *et al.* Social isolation during adolescence induces anxiety behaviors and enhances firing activity in BLA pyramidal neurons via mGluR5 upregulation. *Mol. Neurobiol.* **55**, 5310-5320 (2018).
